# Identification and Genomic Characterization of Two Novel Hepatoviruses in Shrews from Yunnan Province, China

**DOI:** 10.3390/v16060969

**Published:** 2024-06-17

**Authors:** Yi Tang, Kai Zhao, Hong-Min Yin, Li-Ping Yang, Yue-Chun Wu, Feng-Yi Li, Ze Yang, Hui-Xuan Lu, Bo Wang, Yin Yang, Yun-Zhi Zhang, Xing-Lou Yang

**Affiliations:** 1 Yunnan Key Laboratory of Screening and Research on Anti-Pathogenic Plant Resources from Western Yunnan, Key Laboratory for Cross-Border Control and Quarantine of Zoonoses in Universities of Yunnan Province, Institute of Preventive Medicine, School of Public Health, Dali University, Dali 671000, China; tangyiiii@outlook.com (Y.T.); 13099916216@163.com (H.-M.Y.); yangzeyz@163.com (Z.Y.); 2 Key Laboratory of Genetic Evolution & Animal Models, Yunnan Key Laboratory of Biodiversity Information, Kunming Institute of Zoology, Chinese Academy of Sciences, Kunming 650223, China; zhaokai@mail.kiz.ac.cn (K.Z.); yangliping@mail.kiz.ac.cn (L.-P.Y.); wyuechun2022@163.com (Y.-C.W.); lifengyi@mail.kiz.ac.cn (F.-Y.L.); luhuixuan@mail.kiz.ac.cn (H.-X.L.); 3 University of Chinese Academy of Sciences, Beijing 101408, China; 4 Department of Biomedical Sciences and Pathobiology, Virginia Polytechnic Institute and State University, Blacksburg, VA 24060, USA; bowang@vt.edu; 5 Department of Medical, The Second People’s Hospital of Dali Prefecture, Dali 67100, China; yangyin58@163.com

**Keywords:** hepatitis A virus (HAV), *Hepatovirus* (HepV), shrew, next-generation sequencing (NGS), amplicon and QNome nanopore sequencing (A-QNS), codon usage bias, RNA secondary structure, classification, evolution

## Abstract

Hepatitis A virus (HAV), a member of the genus *Hepatovirus* (*Picornaviridae* HepV), remains a significant viral pathogen, frequently causing enterically transmitted hepatitis worldwide. In this study, we conducted an epidemiological survey of HepVs carried by small terrestrial mammals in the wild in Yunnan Province, China. Utilizing HepV-specific broad-spectrum RT-PCR, next-generation sequencing (NGS), and QNome nanopore sequencing (QNS) techniques, we identified and characterized two novel HepVs provisionally named EpMa-HAV and EpLe-HAV, discovered in the long-tailed mountain shrew (*Episoriculus macrurus*) and long-tailed brown-toothed shrew (*Episoriculus leucops*), respectively. Our sequence and phylogenetic analyses of EpMa-HAV and EpLe-HAV indicated that they belong to the species *Hepatovirus I* (HepV-I) clade II, also known as the Chinese shrew HepV clade. Notably, the codon usage bias pattern of novel shrew HepVs is consistent with that of previously identified Chinese shrew HepV. Furthermore, our structural analysis demonstrated that shrew HepVs differ from other mammalian HepVs in RNA secondary structure and exhibit variances in key protein sites. Overall, the discovery of two novel HepVs in shrews expands the host range of HepV and underscores the existence of genetically diverse animal homologs of human HAV within the genus HepV.

## 1. Introduction

The genus *Hepatovirus* (HepV) comprises a group of small, non-enveloped icosahedral picornaviruses containing a single-stranded positive-sense genome ranging from 7200 to 7900 nucleotides (nt) [1]. HepVs have genome organizations with the 5′ ends linked to a small peptide (VPg), a poly(A) tail at the 3′ ends, and a single open reading frame (ORF) flanked by untranslated regions (UTRs) at both ends [2]. The icosahedral capsid of HepVs consists of 60 copies of four structural proteins: VP1, VP2, VP3, and VP4 [3], responsible for host receptor interaction, viral antigenicity, and pathogenicity [3].

Based on the species demarcation criteria, HepV is currently classified into nine species: *Hepatovirus A* (HepV-A) to *Hepatovirus I* (HepV-I) [4]. Primate HepVs belong to HepV-A, representing only a small fraction of the genetic diversity of HepVs [5]. HepV-A genotypes (gt) I–III can infect humans, while genotypes IV–V have been sporadically found in various species of Old World monkeys [6]. In 2015, a study on the surveillance of HepVs in 15,987 specimens from 209 small mammal species worldwide identified 13 novel non-primate HepVs [7]. Additionally, two new species of HepV have been identified in seals (*Phoca vitulina*) and treeshrews (*Tupaia belangeri chinensis*) in the United States [8]. Recently, HepVs have also been found in shrews (*Chodsigoa smithii*), marmots (*Marmota himalayana*), and deer (*Cervus elaphus*) in China [9,10]. According to the genomic characteristics and host differences, the International Committee on the Taxonomy of Viruses (ICTV) has divided non-primate HepVs into eight distinct species: *Hepatovirus B* (PHV) infects harbor seals, *Hepatovirus C* (HepV-C) and *Hepatovirus G* (HepV-G) infect bats, *Hepatovirus D* (HepV-D) and *Hepatovirus E* (HepV-E) infects rodents, *Hepatovirus F* (HepV-F) infects woodchucks, *Hepatovirus H* (HepV-H) infects hedgehogs and treeshrews, and *Hepatovirus I* (HepV-I) infects shrews (https://ictv.global/report/chapter/picornaviridae/picornaviridae/hepatovirus, assessed on 28 April 2024).

Generally, in both non-primate and primate hosts, HepVs exhibit a similar genome organization [11]. Unlike other mammalian picornaviruses, HepV genomes lack a leader (L) protein. The VP2 protein contains YPX_3_L late domain motifs, likely playing a role in quasi-envelope acquisition, along with a predicted transmembrane domain (TM) in the 3A domain and a cis-acting replication element (*cre*) in RNA encoding the 3D^pol^ domain [12,13,14]. Moreover, the VP1 capsid protein of human HAV possesses a carboxy-terminal extension known as pX (sometimes referred to as 2A), implicated in capsid assembly and potentially quasi-envelopment [7,15]. The UTRs at the genome termini vary in length, but suggestive internal ribosome entry site (IRES) structures are present within the 5′ UTR [5]. Notably, the type of IRES may not be conserved across different HepVs [7]. For instance, human HAVs have type III IRESs [16], while certain bat and rodent HepVs contain sequence elements characteristic of type IV IRESs, likely acquired through ancient recombination events involving different viral families [7].

Although diverse HepVs have been reported in different hosts, the evolutionary history of HepVs remains non-conclusive. In this study, we collected 208 samples from small mammals in Yunnan and identified two novel strains of shrew HepVs through next-generation sequencing (NGS) and amplicon-QNome nanopore sequencing (A-QNS). Genomic characterization demonstrates that both strains belong to the novel shrew HepV-I.

## 2. Materials and Methods

### 2.1. Field Samples

The samples were collected in August 2022 from Gongshan County, Nujiang Prefecture in Western Yunnan Province following animal ethics guidelines. Rodents and shrews were captured in cages, euthanized humanely, and their tissues were harvested and stored at −80 °C. All procedures adhered to the guidelines of the Animal Ethics Committee of Dali University (Ethics Number: DLDXLL2020007).

### 2.2. RNA Extraction and Viral Detection

Samples of lung, liver, and intestinal tissues were collected, and the nucleic acids were extracted after thorough grinding using the MagaBio plus Virus DNA/RNA Purification Kit III (Cat. BSC86S1E) according to the manufacturer’s instructions. The one-step nested RT-PCR test was performed using the TIANGEN FastKing One-Step RT-PCR Kit (Cat. KR123) under the following conditions. The first round of amplification: initial reverse transcription at 42 °C for 30 min, 35 cycles of denaturation at 94 °C for 30 s, annealing at 51 °C for 30 s, extension at 72 °C for 30 s, and a final extension at 72 °C for 5 min. The second round of amplification: 95 °C for 3 min before denaturation, 95 °C for 15 s of denaturation, an annealing temperature of 50 °C for 15 s, and 72 °C for 20 s of extension; those three steps for 35 cycles, then 72 °C for extension for 5 min. The primer sequences were as follows: HAV-F1089: GAGATATAYACWTATGCIAGATTTGG, HAV-R1481: CTRAATTCRTTICTCATCATYTGTG, and HAV-R1544: GACATYTTIGCYCTIGCATCYTC [7,17]. Each small mammal species was identified by analyzing the external morphological characteristics and mitochondrial cytochrome b (Cyt-b) gene sequences [18,19,20]. The primer sequences for Cyt-b amplification were: Cytb-F: ATGATATGAAAAACCATCGTTG and Cytb-R: TTTCCNTTTCTGGTTTACAAGAC. Both viral and host amplicons were sequenced by Sangon Biotech Co., (Kunming, China).

### 2.3. Next-Generation Sequencing (NGS) of HepV-Positive Samples

Two individual HepV-positive samples (GS63 and GS159) underwent virome sequencing using a MGI RS2000 sequencer at the BGI Company (Shenzhen, China). Raw reads were subjected to quality control using FastQC version 0.11.8 and Trimmomatic version 0.39 to filter out low-quality reads and remove adaptor sequences. The assembly of high-quality reads utilized Megahit and Genious Primer version 2023.0.1. The obtained contigs were then aligned to a cohort of published non-primate HepV genomes.

### 2.4. Amplicon and QNome Nanopore Sequencing (A-QNS)

A set of amplicon primers was designed for EpLe-HAV-GS159, which had poor coverage by NGS sequencing (Table 1). The first round of amplification conditions was an initial reverse transcription at 55 °C for 30 min and denaturation at 94 °C for 3 min, followed by 40 cycles of denaturation at 94 °C for 30 s, annealing at 55 °C for 30 s, extension at 72 °C for 40 s, and a final extension at 72 °C for 7 min. The second round of amplification conditions were denaturation at 94 °C for 3 min, followed by 40 cycles of denaturation at 94 °C for 15 s, annealing at 52 °C for 30 s, extension at 72 °C for 40 s, and a final extension at 72 °C for 7 min. PCR amplification products underwent agarose gel electrophoresis and recovery with the GelExtraction Kit (Cat. D2500-02). Product concentrations were determined using the Invitrogen dsDNA HS assay Kit (Cat. Q32851) and Qubit nucleic acid quantification instrument. A viral DNA library was prepared according to a commercialized library building kit (Cat. QDL-E V1.1), purified using the Invitrogen dsDNA HS assay Kit, and then mixed with buffer solution from a commercialized sequencing kit (Cat. QDS V1.1) and added to the sequencing chip. After applying voltage, the sample library was captured through nanopores on the detection channel for sequencing.

### 2.5. Rapid Amplification of cDNA Ends (RACE)

5′/3′ RACE amplification was performed for EpLe-GS63-HAV and GS159-HAV using the HiScript-TS 5′/3′ RACE Kit (Cat. RA101). The gene-specific primers used during the 5′/3′ RACE of GS63 and GS159 are listed in Table 1. The conditions for the first round of amplification were as follows: initial denaturation at 98 °C for 30 s, followed by denaturation at 98 °C for 10 s, annealing at 62 °C for 10 s, extension at 72 °C for 45 s for 30 cycles, and a final extension at 72 °C for 5 min. The second round of amplification conditions were initial denaturation at 98 °C for 30 s, denaturation at 98 °C for 10 s, annealing at 58 °C for 10 s, extension at 72 °C for 45 s for 35 cycles, and a final extension at 72 °C for 5 min. The amplified PCR product underwent agarose gel electrophoresis, followed by recovery using the Gel Extraction Kit (Cat. D2500-02), and then sent for sequencing. After gap filling and the completion of 5′ and 3′ RACE, two complete HepV genomes were obtained and deposited in the GenBank database under accession numbers PP723873 and PP723874. Geneious Prime version 2023.0.1 was employed to search for coding sequences (CDSs) in the two complete HepV genomes.

### 2.6. Establishment of qRT PCR Standard Curves

Quantitative Real-Time PCR (qRT PCR) on EpLe-GS63-HAV and GS159-HAV was performed using the HiScript II One Step qRT PCR SYBR Green kit (Cat. Q221). The gel products amplified by EpMa-HAV-GS63 and EpLe-HAV-GS159RT-PCR (the concentrations were 2.75 ng/µL and 108 ng/µL, respectively) were used as the standard samples for the establishment of standard curves. We performed six consecutive dilution gradients on the standard sample, calculated the logarithmic value of the virus copy number for each dilution gradient using a formula as the x-axis of the standard curve, and plotted the obtained Ct value as the y-axis (Figure S1).

### 2.7. Bioinformatic Analysis

The partial VP2 gene and Cyt-b segment were subjected to online Basic Local Alignment Search Tool (BLAST) analysis using NCBI’s Blast tool to identify sequence identities. Coverage statistics for NGS and A-QNS were generated using IGV (Integrative Genomics Viewer) [21]. The assembly of full-length HepV sequences was performed by Geneious Prime version 2023.0.1. Maximum likelihood phylogenetic trees of the partial VP2 (313 bp) and full-length genomic nucleotide sequence were constructed based on alignments using Molecular Evolutionary Genetics Analysis (MEGA) version 10.2.6, with 1000 bootstrap replicates [22]. The aligned dataset was analyzed and a phylogenetic tree constructed using the maximum likelihood (ML) method. Based on the lowest BIC (Bayesian Information Criterion) score, the optimal nucleotide substitution model was determined. Based on the lowest BIC (Bayesian Information Criterion) score, the General Time Reversible model and Gamma Distributed With Invariant Sites (GTR + G + I) were selected as the optimal models for tree construction. Simplot version 3.5.1 was used for recombination and similarity analyses [23,24]. Neighbor-joining phylogenetic trees of the complete genome, polyprotein, P1, 2C + 3CD, VP1, and 3CD were constructed based on the alignments of the nucleotide and amino acid sequences using MEGA version 10.2.6, with 1000 bootstrap replicates. All trees were edited using FigTree version 1.4.4. The structure model of VP1, VP2, and VP3 was predicted using the SWISS-MODEL online tool [25], utilizing the structure of the related PDB protein (PDB: 5WTE) as a template. Predicted protein secondary structures across sequence alignments were compared using ESPript version 3.0. Internal ribosome entry sites (IRESs) were folded manually and using Mfold (RNA Folding Form V2.3). Prediction of the elements was made using Mfold. Transmembrane domains were predicted using TMHMM-2.0. The G + C content, ENCs, and GC3 usage were determined using CodonW and CAIcal [26].

## 3. Results

### 3.1. Routine Laboratory Testing Results

**Figure 1:**
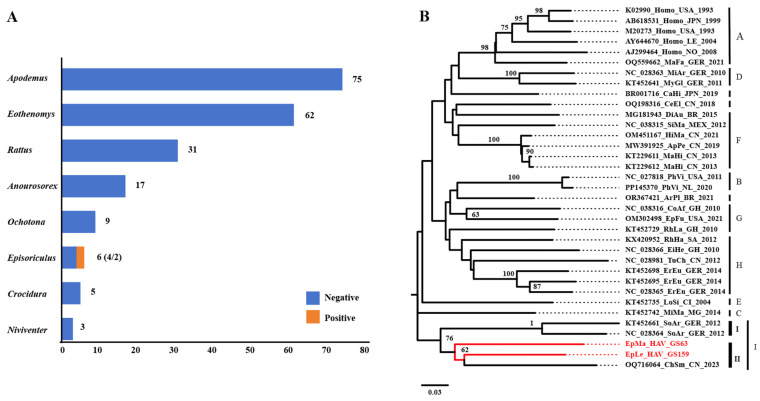
HepV detection, species identification, and virus classification. (**A**) HepV detection in different animal hosts in Yunnan Province, China. HepV-negative and -positive samples are indicated in blue and orange, respectively. (**B**) Maximum likelihood phylogenetic tree of the partial VP2 (313 bp) of two novel shrew HepVs and representative known HepVs. The novel shrew HepVs discovered in this study are presented in red (unmarked as unclassified *Hepatovirus*).

A total of 208 small mammals from eight genera were collected in Yunnan Province, China, and HepV was detected in mixed grinding samples of frozen lung, liver, and intestinal tissues. Among the six samples collected from the genus *Episoriculus*, GS63 and GS159 tested positive for HepV (Figure 1A). Host identification was initially confirmed based on morphological identification in the field by an experienced veterinarian. For HepV-positive samples, host identification was conducted through molecular techniques of Cyt-b amplification [27,28]. Consequently, the hosts of the two HepV-positive samples, GS63 and GS159, were identified as long-tailed mountain shrew (*Episoriculus macrurus*) and long-tailed brown-toothed shrew (*Episoriculus leucops*), respectively. Phylogenetic analysis based on the nucleotide sequences of a partial VP2 gene segment (313 bp) indicated that both samples were closely related to the tentative species HepV-I, suggesting that the detected HepVs in GS63 and GS159 were shrew HepVs, closely resembling an unclassified shrew HepV (GenBank accession number: OQ716064) found in Smith’s shrew (*Chodsigoa smithii*) in Hubei Province, China (Figure 1B).

### 3.2. Viral RNA Copies Determination

The standard curve for viral RNA determination was established to quantify the viral genome copies in different tissues (Figure S1A,B). The qRT PCR method was used to detect the liver and kidney of GS63 and GS169, while the lung tissues of GS63 were exhausted for other viral genome amplification. In these tissue samples, the average copy number of HepVsRNA is as follows: GS63 Kidney is 1.72 × 10^9^ copies/g, GS63 Liver is 9.86 × 10^11^ copies/g, GS159 Kidney is 2.56 × 10^6^ copies/g, GS159 Liver is 2.66 × 10^8^ copies/g, and GS159 Lung is 1.53 × 10^9^ copies/g (Figure S1C).

### 3.3. Genome Amplification

**Figure 2:**
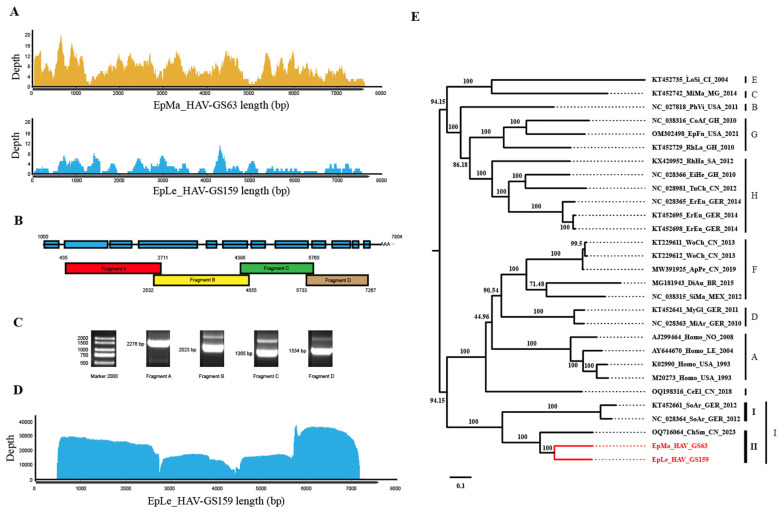
Next-generation sequencing (NGS), amplicon and QNome nanopore sequencing (A-QNS), and the identification of two shrew HepVs. (**A**) NGS reads coverage of the EpMa-HAV-GS63 and EpLe-HAV-GS159 genomes. (**B**) Strategy of the primer design of full-length EpLe-HAV-GS159 genome amplification. (**C**) Gel electrophoresis results of EpLe-HAV-GS159 four amplicons’ enrichment. The discrepancy of fragment size in the gel was caused by different proportions of nucleic acid dye in the DNA ladder and PCR production. (**D**) A-QNS contigs coverage of the EpLe-HAV-GS159 genome. (**E**) Phylogenetic maximum likelihood tree of the full-length genomic nucleotide sequence of EpMa-HAV-GS63 and EpLe-HAV-GS159. Proposed classification of HepV is indicated on the right (the novel shrew HepVs discov-ered in this study are presented in red, unmarked as unclassified *Hepatovirus*).

Next-generation sequencing (NGS) of GS63 and GS159 revealed that GS63 sequencing produced 348 reads, fully covering the entire genome of EpMa-HAV-GS63 (99.45%), except for the 5′ UTR end. However, EpLe-HAV-GS159 sequencing yielded only 112 reads, with 80.01% coverage and multiple gaps (Figure 2A). RACE and Sanger sequencing were employed to complete the sequences at both ends of EpMa-HAV-GS63 and EpLe-HAV-GS159. Due to multiple gaps in EpLe-HAV-GS159, amplicon primers were designed based on the NGS results for nucleic acid enrichment (Table 1). The entire length was divided into four amplification reactions (Figure 2B,C), followed by QNome nanopore sequencing (QNS). The third-generation sequencing of EpLe-HAV-GS159 yielded a total of 110,327 reads, providing 99.99% coverage of the amplicon fragments A to D, except for the RACE at both ends (Figure 2D).

The full-length genomes of EpMa-HAV-S63 and EpLe-HAV-GS159, obtained through NGS, A-QNS, and RACE, were 7633 bp and 7747 bp in length, respectively, including the polyadenylated tail. A phylogenetic tree of the entire viral genome based on nucleotide sequences revealed that EpMa-HAV-GS63 and EpLe-HAV-GS159 formed a distinct cluster with HepV-I, alongside the shrew HepV strain found in Smith’s shrew (*C. smithii*) from Hubei, China (GenBank accession number: OQ716064), and two shrew HepVs from European shrew (*Sorex araneus*) in Germany (GenBank accession numbers: NC_028364 and KT452661) (Figure 2E).

### 3.4. Genome Characterization of Shrew HepVs

**Figure 3:**
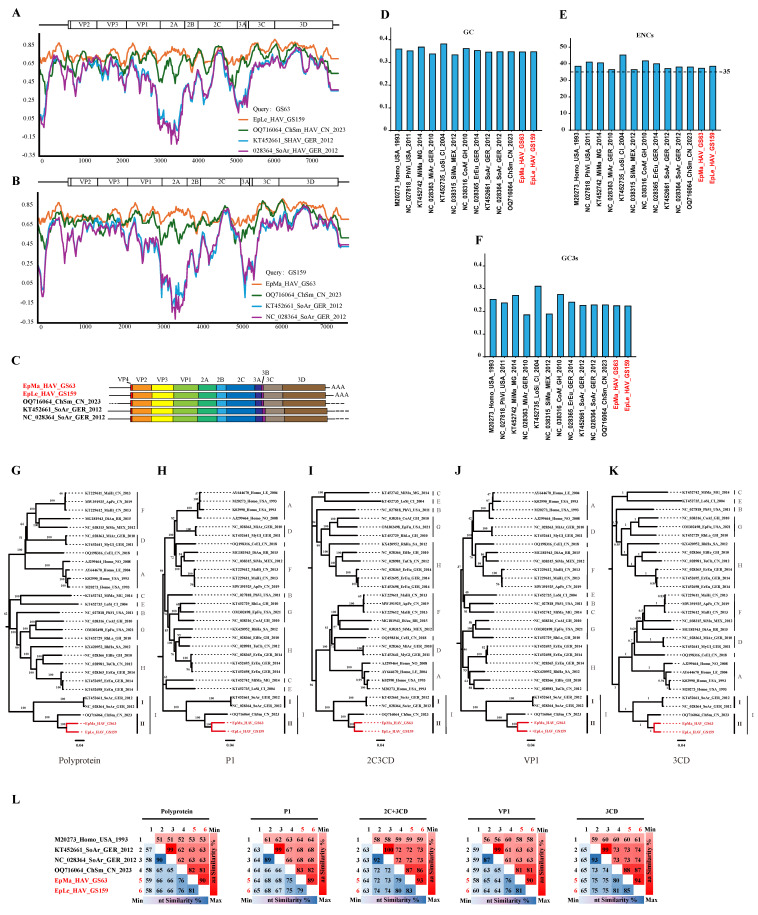
Properties of the genomes of novel shrew HepVs. (**A**) The results of sequences identity analysis on EpMa-HAV-GS63 using Simplot. (**B**) The results of sequences identity analysis on EpLe-HAV-GS159 using Simplot. (**C**) Shrew HepV genome organization. (**D**) Percentage of G + C content of HepV genomes. (**E**) The effective number of codons of HepV genomes. (**F**) The GC content of the third position of the synonymous codon of HepV genomes. (**G**–**K**) Maximum likelihood phylogenetic trees of amino acids for polyprotein (**G**), P1 (VP1–4) (**H**), 2C + 3CD (**I**), VP1 (**J**), and 3CD (**K**). (**L**) amino acid identity of shrew HepVs in polyprotein, P1 (VP1–4), 2C + 3CD, VP1, and 3CD (the novel shrew HepVs discov-ered in this study are presented in red, unmarked as unclassified *Hepatovirus*).

Genomic nucleotide identity analysis indicated that EpMa-HAV-GS63 and EpLe-HAV-GS159, along with the three additional shrew HepVs, were closely related, with the largest nucleotide difference observed in the 2B region (Figure 3A,B). Compared to the previously identified ChSm-HAV in China, the genetic regions with the most considerable differences between EpMa-HAV-GS63 and EpLe-HAV-GS159 were the 2C terminal and the 2A terminal. EpMa-HAV-GS63, EpLe-HAV-GS159, ChSm-HAV, and German shrew HepVs exhibit significant divergence in the 5′ UTR N-end, 2A, and 3A regions (Figure 3A,B).

EpMa-HAV-GS63 and EpLe-HAV-GS159 showed a typical HepV genome structure and contained only one open reading frame (ORF). The EpMa-HAV-GS63 genome consisted of a 728 nt 5′ UTR and an ORF of 6717 nt (encoding a potential polyprotein precursor of 2239 amino acids), followed by a 142 nt 3′ UTR and a poly(A) tail of 46 nt. Similarly, the EpLe-HAV-GS159 genome consisted of a 732 nt 5′ UTR and an ORF of 6723 nt (2241 amino acids), followed by a 261 nt 3′ UTR and a poly(A) tail of 31 nt (Figure 3C). Notably, EpLe-HAV-GS159 differed from EpMa-HAV-GS63 and the other three known shrew HAVs by having a more extended VP4 coding region (Figure 3C).

EpMa-HAV-GS63 and EpLe-HAV-GS159 exhibited their closest genomic nucleotide identity at 79%, significantly lower than the 90% nucleotide identity observed between the German shrew HepVs. Additionally, the genomic nucleotide identity between EpMa-HAV-GS63, EpLe-HAV-GS159, and ChSm-HAV was less than 75%. HepV possesses a low G/C ratio in the genome sequence and a strong codon bias [29]. The GC content of EpMa-HAV-GS63 and EpLe-HAV-GS159 in the ORF genomes was 35.7% and 35.8%, similar to the known shrew HepVs (35.6–35.8%) (Figure 3D). EpMa-HAV-GS63 and EpLe-HAV-GS159 exhibited similar effective numbers of codon (ENC) values (37.68 and 38.90) and GC3 values (0.233 and 0.232) compared to other shrew HepVs (Figure 3E,F).

### 3.5. Amino Acid Difference Analysis

Within the HepV genus, members typically exhibit more than 70% amino acid identity in their polyprotein, P1, and 2C + 3CD [4,11]. Shrew HepVs (HepV-I) share amino acid divergence in polyprotein, P1, and 2C + 3CD higher than 30% compared to other HepVs (Table S1). Both at the nucleotide and amino acid levels, shrew HepVs are distinct from other mammalian HepVs, forming a separate branch. Phylogenetic trees of amino acids for polyprotein, P1 (VP1–4), 2C + 3CD, VP1, and 3CD indicate that EpMa-HAV-GS63 and EpLe-HAV-GS159 belong to clade II (China shrew HepV clade), along with CHSM-HAV of HepV I (Figure 3G–K). The amino acid identities of the polyprotein, 2C + 3CD, and VP1 between HepV-I clade I and clade II were less than 63%, 73%, and 63%, respectively (Figure 3L). Additionally, the HepVs between HepV-I clade I and clade II share 66–68% and 73–74% amino acid identities in P1 and 3CD. The amino acid identities of polyprotein, P1, 2C + 3CD, VP1, and 3CD in clade II were 81~90%, 82~89%, 86~93%, 81~90%, and 87~94%, respectively (Figure 3L).

### 3.6. Secondary RNA Structure of EpMa-HAV-GS63 and EpLe-HAV-GS159

**Figure 4:**
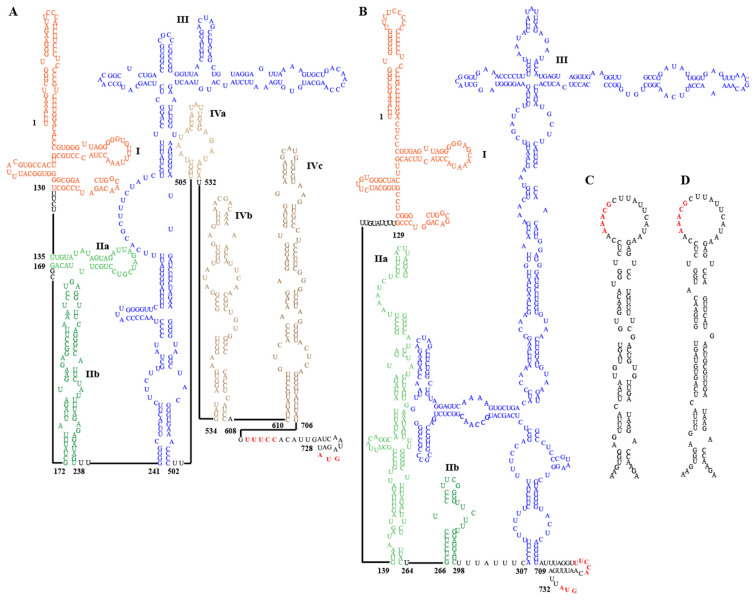
Secondary RNA structure of the 5′ untranslated region (UTR) and cis-acting replication elements (*cre*) of EpMa-HAV-GS63 and EpLe-HAV-GS159. (**A**) Predicted secondary structure of HepV 5′ UTR RNA of EpMa-HAV-GS63. (**B**) Predicted secondary structure of HepV 5′ UTR RNA of EpLe-HAV-GS159. Pictograms represent the full 5′ UTR from the most 5′ nucleotide to the polyprotein initiation codon at the 3′ end. (**C**) Predicted *cre* in the 3D^pol^ genomic region of EpMa-HAV-GS63. (**D**) Predicted *cre* in the 3D^pol^ genomic region of EpLe-HAV-GS159.

Conserved structural elements in the 5′ UTR of most HepVs include several pyrimidine-rich tracts and a large, predicted cruciform stem-loop resembling the type III HAV IRES [16]. However, the 5′ UTRs of viruses from EpMa-HAV-GS63 and EpLe-HAV-GS159 have some distinct characteristics of typical type III IRES (Figure 4A,B). EpMa-HAV-GS63 features a semicircular free loop in the body of the cruciform stem-loop and a small stem-loop structure at the proximal end of the long arm of its cruciform stem-loop (Figure 4A). In contrast, there is a T-shaped stem-loop in the body of the cruciform stem-loop of EpLe-HAV-GS159 (Figure 4B). Both EpMa-HAV-GS63 and EpLe-HAV-GS159 are predicted to have three domains, including domain I, domain II (consisting of two stem-loops: IIa and IIb), and domain III (cruciform stem-loop) (Figure 4A,B). However, EpMa-HAV-GS63 exhibits a domain IV (including IVa–IVc) consisting of three independently connected stem-loops before the initiation codon compared to EpLe-HAV-GS159 (Figure 4A). 

Two important motifs near the 3′ border of the picornavirus IRES were also found in the EpMa-HAV-GS63 and EpLe-HAV-GS159 5′ UTR. The first motif is a UUUCC sequence within the second pyrimidine-rich tract; the second motif is an AUG triplet that functions as an initiation codon in HepVs (Figure 4A,B). Like other HepVs, a putative cis-acting RNA replication element (*cre*) is located near the 5′ end of the 3D^Pol^-coding sequence of EpMa-HAV-GS63 and EpLe-HAV-GS159. It contains a top loop of 18 nt with a stem segment of 25 nt and 28 nt, respectively, and is interrupted by several internal loops and three 1 nt bulges (Figure 4C,D). However, despite the 93.33% nt identity between the *cre* of EpMa-HAV-GS63 and EpLe-HAV-GS159, the similar region in EpMa-HAV-GS63 has one loop more than EpLe-HAV-GS159 (Figure 4C,D). 

**Figure 5:**
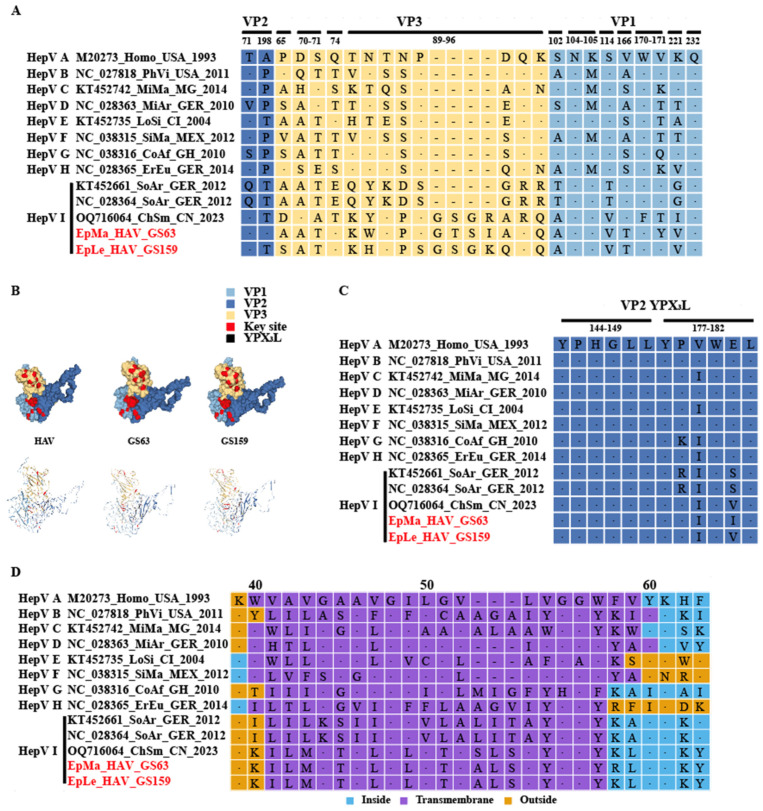
Prediction of HepV epitopes, protein models, and transmembrane domains. (**A**) HepV epitopes associated with neutralization. Background shading and residue color indicate the biochemical properties of residues. (**B**) Predictive protein model of HepV. (**C**) VP2 late domains of HepVs. (**D**) Predicted transmembrane domains of HepVs. (the novel shrew HepVs discov-ered in this study are presented in red).

Human HAV harbors a conformation-dependent immunodominant neutralization site [7,30]. Residues S102, NK104–105, S114, V166, WV170–171, K221, and Q232 of VP1, as well as P65, DS 70–71, and Q74 of VP3, have been implicated in neutralizing epitopes; residues T71 and A198 of VP2 and 89–96 motifs of VP3 may harbor other epitopes (Figure 5A,B and Figures S2–S4). EpMa-HAV-GS63 and EpLe-HAV-GS159 shared 7 of these 13 conformation-dependent immunodominant neutralization site residues (Figure 5A,B). HepV-I clade II shrew HepVs have a four amino acids insertion in HepV 89–96 motifs of VP3 (Figure 5A). The YPX_3_L “late domain” motifs in VP2 that contribute to HepV membrane envelopment by mediating capsid interactions with components of the endosomal sorting complex required for transport (ESCRT) are highly conserved among HepVs (Figure 5B,C) [31].

A transmembrane domain (TM) of 3A, which shares homology with the TM domain of mitochondrial antiviral signaling protein (MAVS), mediates 3ABC precursor colocalization with and cleavage of MAVS, thereby disrupting the activation of IRF3 through the MDA5 pathway [32]. Host-dependent differences in TM exist among HepVs. For example, human HAV and rodent HepVs feature a 3 amino acid deletion at 3A 53–55 compared to HepVs in other types of hosts (Figure 5D). There is also a significant difference in the TM amino acid sequence between clade I and clade II in HepV-I (Figure 5D). Even the TM (ILMGTALGLLTVSLSLYGGY) of CHSM-HAV in clade II differs by one amino acid site from the TM (ILMGTALGLLTVALSLYGGY) sequence of EpMa-HAV-GS63 and EpLe-HAV-GS159 (Figure 5D). Additionally, HepV-E, HepV-F, and HepV-H also exhibit differences with other mammalian HepVs before and after TM, and the predicted protein secondary structure suggested an opposite direction of the protein transmembrane (Figure 5D and Figure S5).

## 4. Discussion

Despite the availability of an effective vaccine, hepatitis A remains a significant public health burden worldwide, with over 150 million new annual infections [33]. According to the data of the ICTV (https://ictv.global/report/chapter/picornaviridae/picornaviridae/hepatovirus, assessed on 28 April 2024) and the World Health Organization (WHO) [33], HAV generally establishes a persistent infection when induced on any of a wide range of primitive cells in vitro, but persistent infection does not occur in vivo, and the viruses are not associated with chronic hepatitis. Human naturally infectious hepatitis is only associated with HepV-A, and there are currently few reports supporting biological data on other HepVs (HepV-B to HepV-I) causing clinical manifestations in their hosts. Over the past decade, there has been a growing recognition of non-primate HAV-related viruses in various animal species such as seals, rodents, shrews, bats, and others [5,7,8,9]. However, research on the detection and distribution of novel HepVs, particularly in regions like China and other biodiversity hotspots in Asia, has been limited, and the evolutionary history of HepV remains unclear. The discovery of EpMa-HAV-GS63 and EpLe-HAV-GS159 provided molecular evidence for new shrew HepVs in China and expanded the host range and geographical distribution of shrew HepVs.

In this study, we investigated 208 small mammals across eight animal genera collected from Nujiang Prefecture, Yunnan Province, in August 2022 to explore the molecular epidemiology of HepV. Consequently, two HepV-positive samples were detected, resulting in a detection rate of 0.96% (2/208). Although slightly higher than the previous HepV detection rate (0.7%, 117/15987) reported for the global HepV surveillance in small mammals [7], the prevalence of HepV varied among different species, with all positive samples in our study originating from shrews. Notably, a previous study on the HepV carried by small mammals worldwide suggested that HepV may have originated in small insectivores and undergone multiple major host transfers during its evolution, such as the possibility of human HAV originating from rodent HepV spillover [7].

The complete genome sequence of EpMa-HAV-GS63 was obtained through the NGS technique, while the full-length genome sequence of GS159 was not obtained due to the low viral load in the sample. Amplicon sequencing, as a complement to NGS, is increasingly utilized for discovering pathogen-specific genomics in cases of low viral load [34,35]. In this study, we established and validated the HepV A-QNS method for the first time and successfully obtained the high-quality, full-length genome sequence of EpLe-HAV-GS159 using A-QNS and RACE techniques. We confirmed that both EpMa-HAV-GS63 and EpLe-HAV-GS159 are typical shrew HepVs belonging to species HepV-I. Based on the full-length genomic sequence analysis, it was determined that EpMa-HAV-GS63 and EpLe-HAV-GS159 belong to HepV-I clade II, along with the previously identified ChSm-HAV, which is distinct from HepV-I clade II from Chinese shrew and HepV-I clade I from German shrew. The amino acid identities of the polyprotein, 2C + 3CD, and VP1 between HepV-I clade I and clade II were less than 63%, 73%, and 63%, respectively (Figure 3L). Shrew HepVs of HepV-I clade I typically exhibit characteristics of type III IRES. In clade II, EpMa-HAV-GS63 and EpLe-HAV-GS159 display significant divergence, including the presence of a semicircular free loop and a T-shaped stem-loop in the body of the cruciform stem-loop. A conserved RNA structure (*cre* element) was found in EpMa-HAV-GS63 and EpLe-HAV-GS159, located near the 5′ end of the 3D^Pol^ region. It also exists in other HepVs and the remotely related avian encephalomyelitis virus [32]. The AAACA/G motif, serving as the template for the uridylation of VPg through a slide-back mechanism, was also identified in EpMa-HAV-GS63 and EpLe-HAV-GS159 [32]. These findings suggest that EpMa-HAV-GS63 and EpLe-HAV-GS159 may process replication mechanisms and tissue tropism similar to those of other known HepVs. However, further studies are warranted to investigate the subtle differences in the secondary structure of the 5′ UTR among EpMa-animal HepVs.

We noticed significant differences between EpMa-HAV-GS63 and EpLe-HAV-GS159 and other HepVs in the 2A–2B and 3A regions. The HepV 2A–2B domain is a genomic hotspot potentially associated with host adaptation and prone to acquiring exogenous sequence elements [7]. HepV 3A shares homology with the TM of host MAVS, a crucial component of virus-activated signaling pathways that trigger protective interferon (IFN) responses [32]. We observed host-dependent differences in the 3A of HepV from various host sources, particularly in the putative TM. The 3A region of Chinese shrew HepVs appears to be distinct from that of German shrew HepVs and other animal HepVs. Additionally, by analyzing the key sites of receptor binding or viral conformation-dependent immunodominant neutralization in VP1, VP2, and VP3, we found that EpMa-HAV-GS63 and EpLe-HAV-GS159 exhibited a similar spatial structure of the VP1-3 tri-protein complex with human HAV. Interestingly, there was a four amino acid fragment insertion at VP3 89–96 aa, which precise function is yet to be determined. Multiple substitutions in the unit points of VP1 and VP2 may cause subtle changes in spatial conformation. The impact of these variances on viral host adaptation needs to be confirmed through additional infection experiments.

Shrew HepVs (HepV-I) exhibit amino acid identity in the polyprotein, P1, and 2C + 3CD, which is less than 30% compared to other mammalian HepVs [11]. This suggests that shrew HepVs may constitute a single species within the genus *Hepatovirus*, according to the species demarcation criteria of *Hepatovirus* from the ICTV. Considering the currently limited HepV sequences in shrews, it is premature to make assumptions about their taxonomic classification. Future research on HepV could prioritize studying shrews to reveal the connection between HepVs found in shrews and other mammals. This scenario parallels that of shrew hantavirus species *Thottimvirus* and other virus species of the genus *Orthohantavirus* [36]. The divergence of members among different HepV species ranges from 0.18 to 0.40 for P1 and 0.19 to 0.49 for 3CD [11]. Although the amino acid differences between P1 and 3CD viruses within clade II were smaller than those typically used for HepV interspecific classification criteria, their host animals were different. Despite originating from different species within the same study area and exhibiting amino acid similarities exceeding 81%, EpMa-HAV-GS63 and EpLe-HAV-GS159 display significant disparities in certain crucial regions. These differences include variations in the secondary structure of 5′ UTR, the length of VP4, and distinctions in the neutralization of VP1–3 and associated key sites. This suggests that EpMa-HAV-GS63, EpLe-HAV-GS159, and CHSM-HAV represent three distinct proximal shrew HepVs.

## 5. Conclusions

The discovery of two novel shrew HepVs (EpMa-HAV-GS63 and EpLe-HAV-GS159) in Yunnan Province, China, is significant, as it indicates the presence of genetically diverse HepVs in different shrew species. By comparing the newly discovered and reported shrew HepVs with other mammalian HepVs, we firmly confirm that the HepVs carried by shrews belong to a new species HepV-I of the genus *Hepatovirus* that is evolutionarily close to human HAV. The high-quality genomes of these two novel shrew HepVs obtained for the first time by NGS and A-QNS undoubtedly aid in understanding the evolutionary history of human HAV.

## Figures and Tables

**Table 1:** Primers used for the complete genome sequencing of EpLe-HAV-GS159 and 5′/3′ rapid amplification of cDNA ends (RACE) and fragment amplification and the quantification of EpMa-HAV-GS63 and EpLe-HAV-GS159.

Virus Targeted	Primer	Sequence (5′-3′)	Use
EpLe-HAV-GS159	GS159-HAV1-F1	ACTGAAACTGGTAAGCAATGTCG	Nested RT-PCR for full-genome sequence of EpLe-HAV-GS159
	GS159-HAV1-R1	AACCCAATAGTTACCAGTTGCC	
	GS159-HAV2-F1	TCATTGGTCAGAGCTTCAAATG	
	GS159-HAV2-R1	TCCCAATAATCACCAGAAACAGG	
	GS159-HAV3-F1	AGATTGTATTTCTAGAGTGCATC	
	GS159-HAV3-R1	ATGCTGGACCAATAGTCAATTC	
	GS159-HAV4-F1	CACTGGTCACCTCCTGTCAA	
	GS159-HAV4-R1	AGATTCTGAACAAACTCAGCATC	
	GS159-HAV2-F2	ACTGATGTAGATGGATTGATCTGG	
	GS159-HAV2-R2	TGGTTCAGGTCTCACAACAGC	
	GS159-HAV3-F2	AGCTGTTGTGAGACCTGAACC	
	GS159-HAV3-R2	ACTGACCCATCATCTTTCTTGTG	
	GS159-HAV4-F2	TGATGATTTCTGAAGGTGCC	
	GS159-HAV4-R2	TTCTCAGAGATTGCAGGCC	
EpLe-HAV-GS63	GS63-5′-R1	TCACCCGTAGCCTACCCCTTCTAGAAG	5′ RACE of EpMa-HAV-GS63
	GS63-5′-R2	AGATCGACATTGCTTACCAGTTTCAG	
EpLe-HAV-GS159	GS159-5′-R1	TAGCCTACCCCTTCTAGAAGATCGAC	5′ RACE of EpLe-HAV-GS159
	GS159-5′-R2	AGATCGACATTGCTTACCAGTTTCAG	
	GS159-5′-F1	AGTGCCTAAGGTTGTGCCTGTG	3′ RACE of EpLe-HAV-GS159
	GS159-5′-F2	AGGGCGTGATTAGGCCTGCAATC	
EpLe-HAV-GS63	GS63-HAV-1F	ACCTACCCTGCGTTCACCGTG	RT-PCR for part-genome sequence of EpLe-HAV-GS63
	GS63-HAV-1R	TCTCATCATCTGAGTCCAGACAG	
EpLe-HAV-GS159	GS159-HAV1-F1	ACTGAAACTGGTAAGCAATGTCG	RT-PCR for part-genome sequence of EpLe-HAV-GS159
	GS159-GAP1-R1	ACCTGACTCCTCATCACCAG	
EpLe-HAV-GS63	GS63-qP-F	AAGGTAGTGTTCATGCAGCAG	qPT-PCR for EpMa-HAV-GS63
	GS63-qP-R	TGAGTCTTTCGAGAACCAGGC	
EpLe-HAV-GS159	GS159-qP-F	ATGAGTAGGTGAAGGTTGCCG	qPT-PCR for EpLe-HAV-GS159
	GS159-qP-R	TTACACCAAGCATCCCTCCAC	

## Data Availability

The datasets analyzed during the current study are available from the corresponding authors on reasonable request. All the sequences in this manuscript can be obtained from the NCBI database (https://www.ncbi.nlm.nih.gov, accessed on 13 June 2024).

## Supplementary Materials

The following supporting information can be downloaded at https://www.mdpi.com/article/10.3390/v16060969/s1, Figure S1: establishment of qRT PCR standard curves and viral RNA copies determination; Figure S2: Identification of VP1 structural elements; Figure S3: Identification of VP2 structural elements; Figure S4: Identification of VP3 structural elements; Figure S5: Predicted transmembrane domains of HepVs; Table S1: Pairwise comparisons of the similarity of the EpMa_HAV_GS63 and EpLe_HAV_GS159 with those of other, representative members of the genus *Hepatovirus* in full length genome, polyprotein, P1, 3CD, VP1, 2C + 3CD.
